# Machine Learning Algorithm for the Detection of Tumor Microsatellite Instability Based on Multiomics Biomarkers

**DOI:** 10.1200/CCI-25-00367

**Published:** 2026-06-25

**Authors:** Kyle C. Strickland, Zachary D. Wallen, Sarabjot Pabla, Heidi C. Ko, Rebecca A. Previs, Michelle F. Green, Stephanie Hastings, Alicia Dillard, Pratheesh Sathyan, Kamal S. Saini, Taylor J. Jensen, Brian J. Caveney, Marcia Eisenberg, Shakti Ramkissoon, Eric A. Severson

**Affiliations:** ^1^ Labcorp, Durham, NC; ^2^ Duke University Medical Center, Duke Cancer Institute, Department of Pathology, Durham, NC; ^3^ Labcorp, Buffalo, NY; ^4^ Duke University Medical Center, Duke Cancer Institute, Department of Obstetrics & Gynecology, Division of Gynecologic Oncology, Durham, NC; ^5^ Illumina Inc, San Diego, CA; ^6^ Fortrea Inc, Durham, NC; ^7^ Labcorp, Burlington, NC; ^8^ Wake Forest Comprehensive Cancer Center and Department of Pathology, Wake Forest School of Medicine, Winston-Salem, NC

## Abstract

**PURPOSE:**

Accurate classification of microsatellite instability (MSI) in advanced cancers is critical for identifying patients who may benefit from immune checkpoint inhibitors. However, variability in MSI detection workflows can lead to missed MSI-high cases, indicating need for complementary screening approaches. Using next-generation sequencing (NGS) data from colorectal tumors, we developed a machine learning (ML) model to predict MSI status using immune-related gene expression profiles and pathogenic single-nucleotide variants (SNVs) and copy-number variants (CNVs).

**MATERIALS AND METHODS:**

We analyzed NGS data from 2,756 patients with colorectal cancer (CRC), including DNA panel results for SNVs and CNVs, RNA sequencing of immune-related genes, and tumor mutation burden (TMB). ML algorithms were trained on 70% of the CRC cohort using TMB and selected features by Boruta algorithm. Trained models were tested on the remainder of the CRC cohort and The Cancer Genome Atlas (TCGA) colorectal (COAD) and rectal (READ) adenocarcinoma data sets. To assess the translatability to other cancer types, uterine and gastric cancer cases were tested.

**RESULTS:**

Feature selection identified 107 features for model training, including SNVs and CNVs. The CART model with the highest mean accuracy, precision, and recall showed strong performance across the CRC, TCGA COAD/READ, uterine, and gastric cancer cohorts, ranging from 78% sensitivity in uterine cancer to 99%-100% specificity and negative predictive value in CRC. Of the 53 indeterminate CRC and uterine cases, 15% were classified as likely MSI-high. Of these, 75% had mismatch repair immunohistochemistry results available, with 83% showing MLH1 and PMS2 loss.

**CONCLUSION:**

Our ML approach accurately predicted MSI status in colorectal and uterine cancers using multiomics data derived from NGS, without relying on direct microsatellite sequencing. The ability to identify MSI-high tumors among indeterminate cases demonstrates potential to improve diagnostic precision and ensures timely access to immunotherapy for patients with MSI-high disease.

## INTRODUCTION

Microsatellite instability (MSI) is a genomic hallmark of defective DNA mismatch repair (MMR), characterized by length alterations in short tandem repeats. MSI is most commonly observed in colorectal, endometrial, and gastric cancers, and is associated with a high neoantigen load that promotes immune recognition.^1,2^ Consequently, MSI-high (MSI-H) tumors are particularly responsive to immune checkpoint inhibitors (ICIs), often resulting in improved clinical outcomes.^3-5^

**CONTEXT**

**Key Objective**
Can microsatellite instability (MSI) status be accurately inferred from multiomics next-generation sequencing (NGS) data without direct microsatellite analysis, particularly in cases with indeterminate MSI testing results?
**Knowledge Generated**
A machine learning model integrating immune gene expression, tumor mutational burden, and genomic alterations was able to accurately classify MSI status across colorectal, uterine, gastric, and The Cancer Genome Atlas cohorts. Among tumors with indeterminate microsatellite sequencing, the model identified a subset as likely MSI high, many of which demonstrated mismatch repair protein loss by immunohistochemistry.
**Relevance *(P.P. Yu)***
A machine-learning algorithm to predict MSI status may have clinical relevance in certain patients.*
*Relevance section written by *JCO Clinical Cancer Informatics* Associate Editor Peter Paul Yu, MD, FACP, FASCO.

Accurate MSI detection is essential for identifying patients likely to benefit from ICI therapy. Conventional methods, including immunohistochemistry (IHC) and polymerase chain reaction (PCR)–based analysis of microsatellite loci, are widely used but can be limited by technical challenges. The repetitive nature of microsatellite sequences complicates amplification and alignment, leading to sequencing artifacts and interpretive difficulties.^6^ Additionally, high mutation rates in these regions further complicate the alignment and interpretation of sequencing data.^6^ These issues are exacerbated in tumors with atypical MMR phenotypes or low tumor purity.

Next-generation sequencing (NGS) enables broad biomarker profiling, including MSI, tumor mutational burden (TMB), single-nucleotide variants (SNVs), copy-number alterations, and immune gene expression across hundreds of genes simultaneously.^7^ Due to the complexity of microsatellite sequencing, computational algorithms such as MSIsensor2, MiMSI, MSINGB, and Foundation Medicine's fraction-based method are commonly used to aid MSI detection when evaluating NGS data.^8-11^ Although these tools generally perform well, they may still yield indeterminate results in a small minority of cases, prompting the need for orthogonal testing.

We hypothesized that MSI status could be inferred without direct evaluation of microsatellite loci, by leveraging broader genomic and transcriptomic signals captured through NGS. Here, we report the development of a machine learning (ML) model that predicts MSI status using multiomics biomarkers derived from genomic alterations and immune gene expression data. This manuscript details the model's performance and explores its potential clinical utility in guiding immunotherapy decision making.

## MATERIALS AND METHODS

### Internal Cohorts

Approval for this study, including waiver of informed consent, was obtained from the Western Institutional Review Board Copernicus Group (WCG protocol No. 1340120). We queried our internal database for colorectal cancer (CRC) cases evaluated by NGS via the OmniSeq INSIGHT assay (Labcorp, Buffalo, NY) from June 2021 to January 2025. Both DNA and RNA sequencing were performed as a standard part of the OmniSeq INSIGHT clinical assay for all cases in the internal cohorts. Cases passing microsatellite NGS were randomly divided into training (70%) and testing (30%) cohorts for feature selection, training, and validation of ML classification models. To investigate the performance of models on different tumor types with a high prevalence of MSI, we evaluated cohorts of uterine and gastric cancer cases tested at Labcorp using the same NGS-based assay and within the same time frame as the CRC training and testing cohorts. Data for cases of colorectal and uterine cancer with partial microsatellite NGS results but an indeterminate MSI status due to low microsatellite coverage (n = 53) were also extracted from the CRC and uterine cohorts for proof-of-concept testing of the top-performing ML model.

### The Cancer Genome Atlas Data Set

We obtained publicly available data from the Cancer Genome Atlas (TCGA) for colon adenocarcinoma (COAD) and rectal adenocarcinoma (READ) to use as an independent validation data set for the trained models.^12-14^ MSI status, clinical, and immune gene expression data for TCGA cases were obtained from the Genomic Data Commons (GDC) Data Portal.^15^ MSI data was downloaded using the TCGAbiolinks R package v 2.34.0,^16^ while clinical and gene expression data were downloaded from the HTML portal interface. Both “TCGAquery_subtype” and “PanCancerAtlas_subtypes” functions were used to download MSI data as each downloaded slightly different TCGA cases, then unique results from both queries were collated together. Designations of MSI-low were classified as unknown, leaving only microsatellite stable (MSS) and MSI-H designations.

### Genomic Profiling

For internal cases, NGS testing was performed as previously described.^7,17^ Briefly, formalin-fixed, paraffin-embedded tissue blocks of tumor specimens were sectioned and evaluated by hematoxylin and eosin (H&E) staining to confirm and select adequate tumor content for nucleic acid extraction. DNA and RNA were then co-extracted from the same tissue using automated workflows. DNA was sequenced using the TruSight Oncology 500 assay (Illumina, San Diego, CA) for the detection of somatic alterations in the full exonic coding regions of 523 cancer-related genes, including SNVs, small insertions and deletions, and copy-number variants (CNVs) in 59 selected genes, as well as calculation of TMB (mutations/megabase) and assessment of microsatellite unstable sites to evaluate MSI status. Extracted RNA was sequenced to detect fusions and splice site variants in select genes. Only data for known pathogenic SNVs, CNVs, and TMB were included in the training data for the ML models. Gene fusions were not included to make trained ML models generalizable to external validation data, which did not use RNA-seq for fusion detection. Genomic alteration data were summarized at the gene level for each case by counting the number of SNVs and CNVs detected within a gene, creating a single numeric measure of genomic variant dosage for each gene.

For TCGA cases, DNA extraction from tumor specimens was performed in various resource centers. Isolated DNA was shipped to genomic sequencing centers where whole-exome sequencing on SOLiD (Thermo Fisher, Waltham, MA) or HiSeq (Illumina, San Diego, CA) platforms was performed for the detection of genomic alterations.^12^ TCGA masked somatic mutation data were obtained from the GDC Data Portal using the “GDCquery,” “GDCdownload,” and “GDCprepare” functions from TCGAbiolinks. TMB was calculated from somatic mutation data by dividing the number of variants detected for a tumor sample by 30 (assuming a whole-exome sequencing background of approximately 30 Mb). If a patient had multiple tumor samples, the median TMB value was used as their TMB value. Only exonic variants of class SNV, indel, insertion, and deletion were included in the data used for ML model testing.

### Gene Expression Profiling and Bioinformatics Processing

For internal cases, RNA sequencing was performed using the Oncomine Immune Response Research Assay (OIRRA) to interrogate gene expression of 395 immune-related genes (Thermo Fisher, Waltham, MA). Absolute read counts for each gene transcript were generated using the Ion Torrent Suite Software plugin immuneResponseRNA (Thermo Fisher, Waltham, MA), then background read counts from a sequenced no-template control sample were subtracted to produce background-subtracted read counts. Per-sample normalized reads per million (nRPM) values were then calculated to make RNA-seq measurements across runs and samples comparable. Briefly, sample background-subtracted read counts are normalized by obtaining sample-to-control ratios of 10 housekeeping gene background-subtracted read counts compared against a preconstructed housekeeping gene RPM profile from an external, validated control sample, and the median ratio is used as a normalization ratio for the sample. Then, for each transcript, sample background-subtracted read counts are divided by the normalization ratio to obtain nRPM values. From the nRPM values, normalized gene expression ranks are calculated as a percentile rank from 0 to 100 by comparing nRPM values to those of a pan-cancer reference population derived from 735 unique tumors.

For TCGA cases, whole-transcriptome RNA sequencing was performed; therefore, after extracting whole-transcriptome data from the GDC database, it was filtered for genes targeted by OIRRA and then processed in the same manner as the CRC and uterine cases.

### Feature Selection, Model Training, and Model Evaluation

Feature selection was performed on the CRC training data set with the Boruta feature selection algorithm (Boruta R package v 8.0.0) to determine which immune gene expression and genomic features were most important in differentiating MSI cases from MSS cases.^18^ Feature selection was performed for all gene expression and genomic features at once, and features selected by the Boruta algorithm (along with TMB) were used in training the ML models.

Models assessed in this study included 10 commonly used algorithms: logistic regression, k-nearest neighbor, linear discriminant analysis, support vector machine (SVM) with a radial kernel (SVM radial), random forest, classification and regression trees (CART), XGBoost, multilayer perceptron neural network, naïve Bayes, and gradient boosting machine. Fitting, training, testing, and evaluation of models were performed using the tidymodels R package v 1.2.0 and associated packages. A 10-fold cross-validation schema was used during training. Trained models were then used to predict the MSI status of cases in the CRC testing data set. Model predictions were based on the standard default probability threshold of 0.5. Model predictions of test cases were compared with their MSI status obtained from microsatellite NGS using confusion matrices. Common classification evaluation metrics were then calculated for each model, including sensitivity/recall, specificity, positive predictive value, negative predictive value (NPV), balanced accuracy, and F1. Multinomial parametric bootstrapping with 2,000 iterations was performed to calculate corresponding 95% CIs for each point estimate. Model predictions and evaluations were repeated for the TCGA, uterine, and gastric cohorts. Receiver operating curves and precision-recall (PR) curves were generated, and the AUC was calculated for each cohort using the top-performing model.

### Predicting MSI Status for Cases Failing Microsatellite Testing

We identified CRC and uterine cases with partial microsatellite NGS results but with an indeterminate MSI status due to insufficient microsatellite coverage, so we could use the top-performing model to predict MSI status for these cases. Many cases failing microsatellite NGS also had missing data for genomic or immune gene expression features, so missing data were imputed using the Multivariate Imputation by Chained Equations (MICE) method with predictive mean matching (PMM) as implemented in the mice R package v 3.17.0. Data for MSI indeterminate cases were combined with passing cases to improve the accuracy and robustness of data imputation. Once missing data were imputed, we used the top-performing classification model to predict the MSI status of indeterminate cases, labeling each case as likely MSI-H or likely MSS. If available, pathology reports with IHC results for MSI or MMR and partial microsatellite NGS results were reviewed to provide further confirmation for the ML model–derived MSI status.

### Data and Code Availability

The genomic and transcriptomic data sets used in this study were generated as part of routine clinical testing performed by Labcorp/OmniSeq and contain protected patient health information that cannot be publicly released. In addition, the ML workflow, feature-processing strategy, and associated algorithmic implementation are currently the subject of an active patent application (US Patent Application No. 63/765,274) assigned to OmniSeq, Inc (Labcorp). As a result, the analysis code and raw internal data sets cannot be shared at this time. Deidentified, aggregate-level results supporting the findings of this study are available from the authors upon reasonable request, subject to institutional data-use policies and intellectual property restrictions.

## RESULTS

### Model Testing, Validation, and Evaluation of Performance

**FIG 1.**
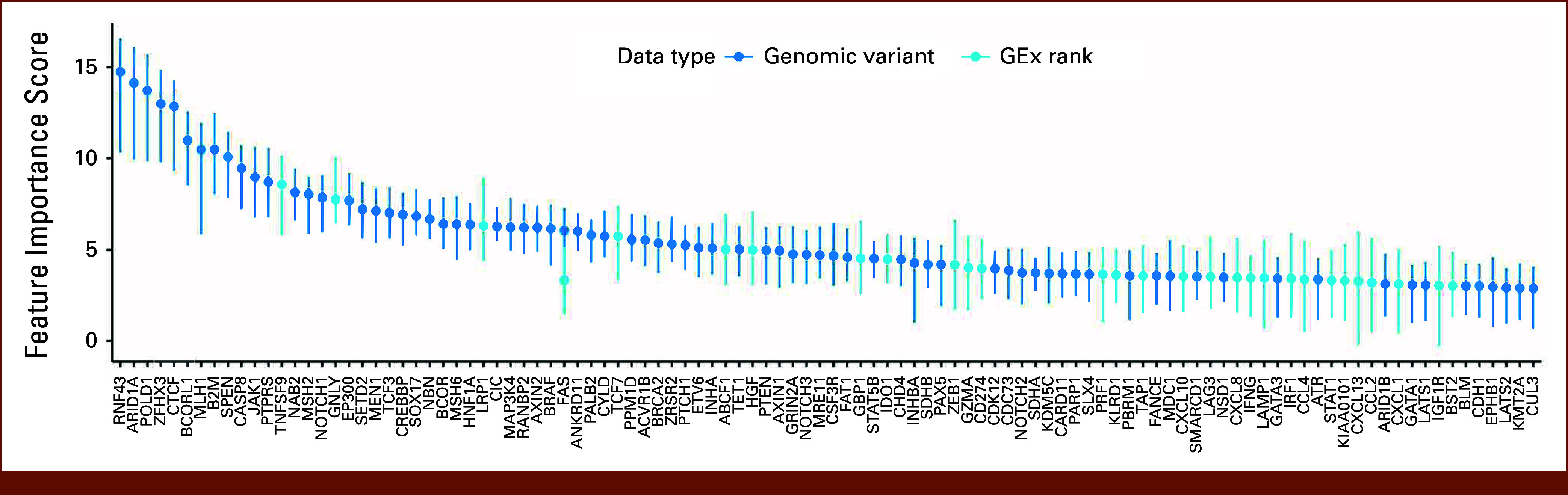
Top-ranked genomic and gene expression features contributing to MSI prediction identified by the Boruta algorithm. Feature importance scores from the machine learning model highlight genomic and transcriptomic variables most predictive of MSI status. Genes such as *RNF43*, *ARID1A*, and *MMR* pathway members (*MSH2*, *MSH6*, and *MLH1*) were among the highest-ranked features. *PMS2* was notably absent, likely due to pseudogene interference from *PMS2CL*, which complicates variant interpretation in exons 11-15. Data types include gene expressions, SNVs, and CNVs. CNV, copy-number alteration; GEx, gene expression; MSI, microsatellite instability; SNV, single-nucleotide variant.

**FIG 2.**
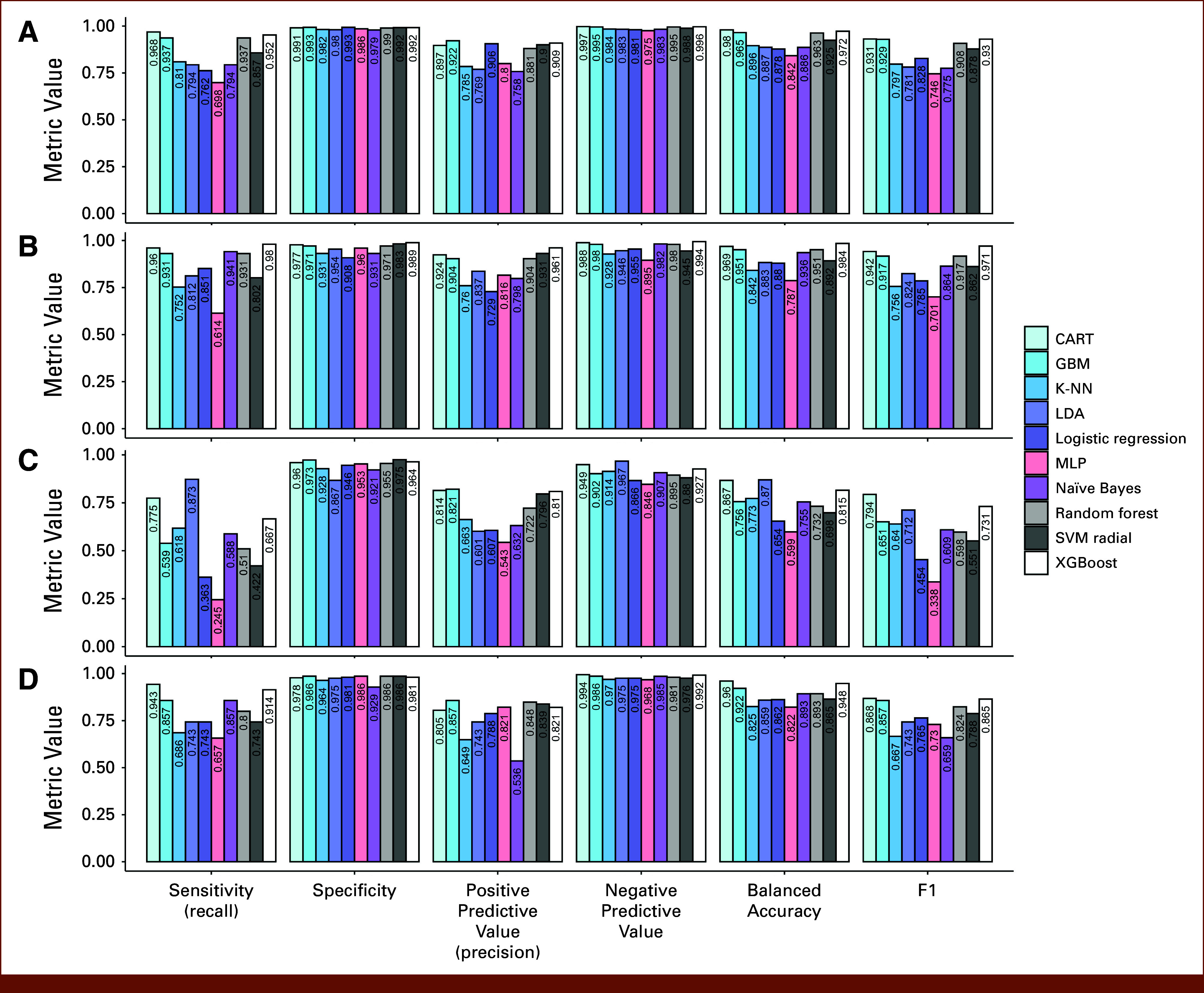
Classification performance metrics for various ML models trained on genomic and immune gene expression data chosen from feature selection. Ten commonly used ML models were trained on genomic and immune gene expression data from an internal CRC training data set and tested on (A) an internal CRC data set, (B) TCGA CRC data set (COAD and READ cases), (C) an internal uterine cancer data set, and (D) an internal gastric cancer data set. The top-performing model was chosen as the highest average between balanced accuracy and F1 score across data sets. CIs of point estimates were not included to help plot clarity, but can be found in the Data Supplement (Table S2). COAD, colorectal adenocarcinoma; CRC, colorectal cancer; ML, machine learning; READ, rectal adenocarcinoma; TCGA, The Cancer Genome Atlas.

**FIG 3.**
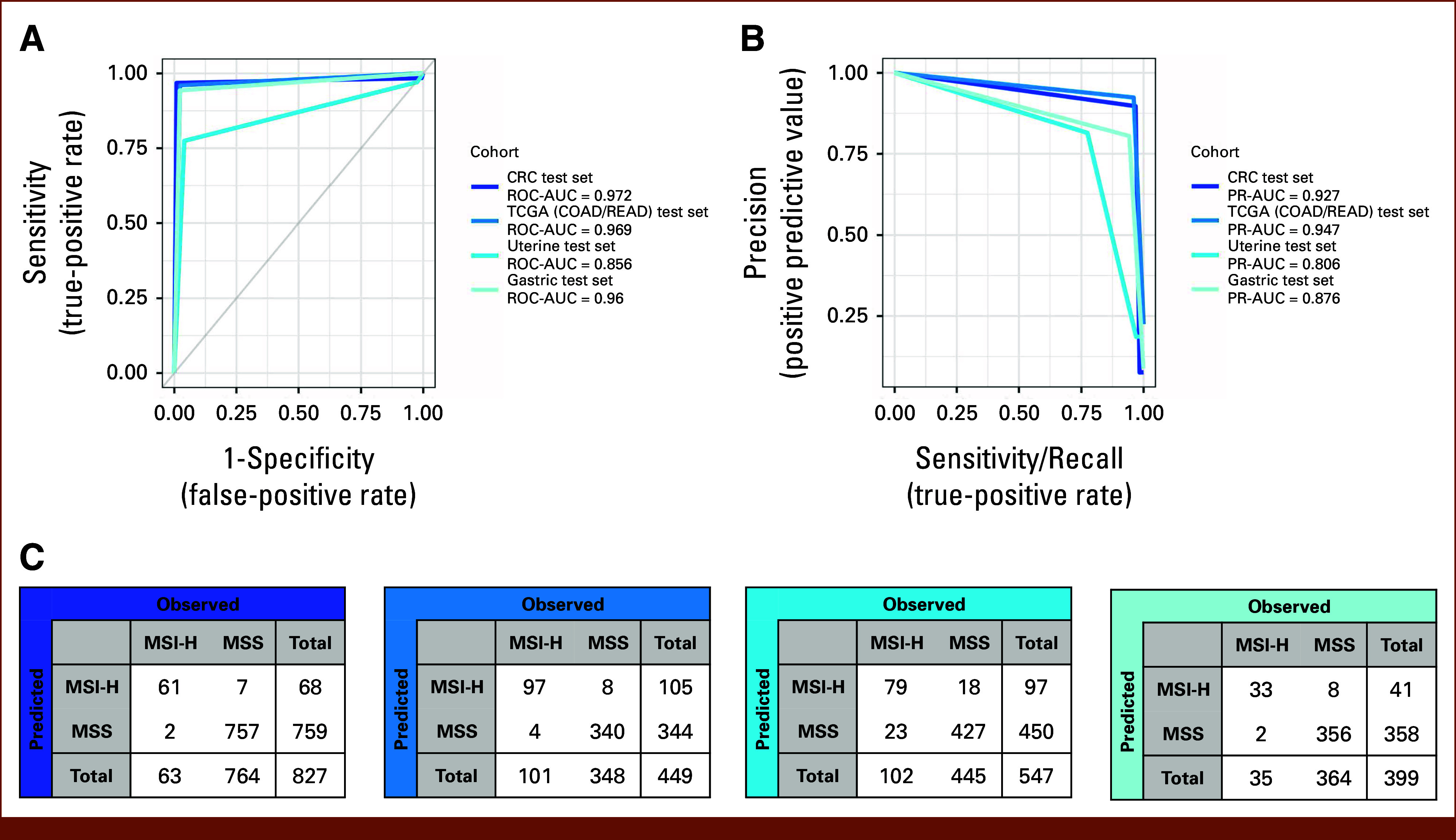
Classification performance of the top-performing ML model, CART. The CART model was chosen as the top-performing machine learning model after testing across data sets. The (A) ROC and (B) PR curves and corresponding AUC scores were plotted and calculated for each testing data set. (C) Confusion matrices for predicted versus observed MSI statuses for each of the testing data sets were constructed to observe the number of correctly classified cases. CART, classification and regression trees; COAD, colorectal adenocarcinoma; CRC, colorectal cancer; MSI, microsatellite instability; PR, precision-recall; READ, rectal adenocarcinoma; ROC, receiver operating characteristic; TCGA, The Cancer Genome Atlas.

We identified 2,756 cases of CRC evaluated by NGS at Labcorp that passed microsatellite NGS (Table 1). Of the passing cases, 70% (n = 1,929) were used for ML model training and feature selection, and 30% (n = 827) were used for testing the trained models and evaluating their performance (Table 1). Using the Boruta feature selection algorithm, we identified 107 features deemed informative for distinguishing MSI-H from MSS tumors, including SNVs and CNVs in 78 genes and the expression of 29 immune genes (Fig 1, Data Supplement, Table S1). These features and TMB were used to train 10 different classification models, which, when tested on the CRC testing cohort, resulted in a wide range of performance metrics from 0.7 (95% CI, 0.6 to 0.8; MLP; sensitivity) to approximately 1 (95% CI, 0.99 to 1; CART; specificity and NPV; Fig 2A, Data Supplement, Table S2). We further assessed model performance on three orthogonal retrospective cohorts: the COAD/READ TCGA cohorts (n = 537) and two internal cohorts of uterine and gastric cancer cases evaluated by the same NGS assay and within the same time frame as the CRC cohort (n = 547 and n = 399, respectively; Table 1, Figs 2B-2D, Data Supplement, Tables S2B-S2D). We found that one model, CART, had the best performance overall, having the highest mean balanced accuracy and F1 measures across cohorts (Fig 2, Data Supplement, Table S2). The CART model exhibited a high degree of predictive accuracy resulting in receiver operating characteristic-AUC metrics of 0.86–0.97 (Fig 3A) and precision recall-AUC metrics of 0.81–0.95 (Fig 3B) depending on the cohort tested. Concordance between CART-derived MSI status and microsatellite NGS results was 98.9%, 97.3%, 92.5%, and 97.5% in the CRC, TCGA, uterine, and gastric testing cohorts, respectively (Fig 3C).

**TABLE 1.** Demographics of Training and Testing Cohorts

Cohort	Sample Size, No.	Median Age, Years	Minimum Age, Years	Maximum Age, Years	Female, %	MSI-High, %
CRC training	1,929	65	22	≥90	46	7
CRC testing	827	65	27	≥90	44	8
TCGA (COAD/READ)	537	69	34	≥90	52	19
Uterine testing	547	69	26	≥90	100	19
Gastric testing	399	69	13	≥90	38	9
Indeterminate CRC and uterine	53	65	40	85	58	11^a^

Abbreviations: CART, classification and regression trees; COAD, colorectal adenocarcinoma; CRC, colorectal cancer; MSI, microsatellite instability; READ, rectal adenocarcinoma; TCGA, The Cancer Genome Atlas.

a Predicted using trained rpart (CART) model.

### Identifying Likely MSI-High Tumors From Cases That Technically Failed Microsatellite Testing

**FIG 4.**
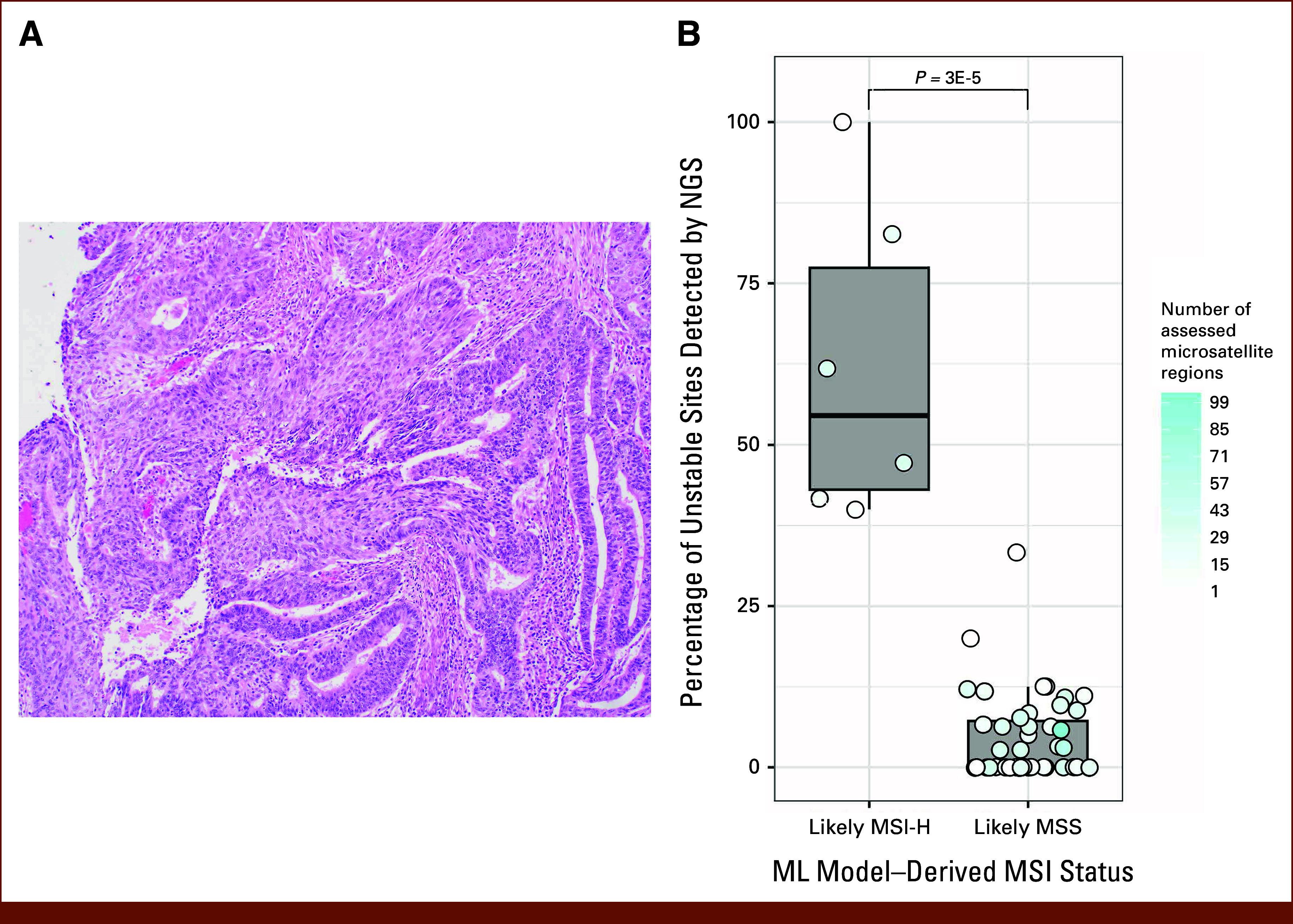
Validation of MSI-H predictions in indeterminate NGS cases. (A) Representative histologic image (H&E-stained, 20× magnification) of a FIGO grade 3 endometrioid endometrial adenocarcinoma. Sequencing was indeterminate for MSI status by NGS due to insufficient microsatellite coverage. This case was flagged as likely MSI-H by the prediction algorithm. Review of the pathology report revealed the tumor exhibited loss of MLH1 and PMS2 by IHC, supporting an MSI-H classification. (B) Box-and-whisker plot showing the percentage of unstable microsatellite loci detected in the setting of insufficient microsatellite coverage, stratified by ML algorithm prediction (likely MSI-H *v* likely MSS). Likely MSI-H cases had significantly higher percentages of unstable loci (*P* = 3E–5), supporting the accuracy of ML-based classification in indeterminate cases. FIGO, Federation Internationale de Gynecologie et d'Obstetrique; H&E, hematoxylin-eosin; IHC, immunohistochemistry; ML, machine learning; MSI, microsatellite instability; MSI-H, MSI-high; MSS, MSI-stable; NGS, next-generation sequencing.

To evaluate MSI status in colorectal and uterine cancer cases with indeterminate microsatellite sequencing results (n = 53), we applied the CART model, which demonstrated strong performance across cohorts (Table 1, Data Supplement, Table S3). These cases lacked sufficient microsatellite coverage for definitive classification but had partial data available from the NGS assay. Among the indeterminate cases, 16 (30.1%) passed both DNA and RNA components, and the remainder had incomplete data: 4 (7.5%) passed DNA testing but failed the RNA component, 21 (39.6%) passed RNA testing but failed the DNA component, and 12 (22.6%) failed both DNA and RNA testing components. To address missing values, we used MICE with PMM across the combined cohort, enabling robust estimation of SNVs, CNVs, and gene expression features. Based on the imputed and available data, the model classified 6 cases (11.3%) as likely MSI-H (Data Supplement, Table S3). Supporting evidence included MMR IHC showing MLH1 and PMS2 loss in 4 (66.7%) of these cases, and partial microsatellite sequencing revealing ≥40% unstable loci in all 6 (100%). As an example, one was a Federation Internationale de Gynecologie et d'Obstetrique (FIGO) grade 3 endometrioid endometrial adenocarcinoma with reported loss of MLH1/PMS2 by IHC and partial microsatellite sequencing showing instability in 5 of 12 (41.7%) interpretable microsatellite sites (Fig 4A). Of cases 47 classified as likely MSS, 20 (42.5%) had MMR IHC results, of which 19 (95%) showed intact MMR protein expression and 1 (5%) case demonstrated loss of MLH1 and PMS2 by IHC. None (0%) of the likely MSS indeterminate cases exhibited >20% unstable loci in partial microsatellite analysis. Overall, likely MSI-H cases had significantly higher percentages of unstable microsatellite sites compared with likely MSS cases (*P* = 3E–5; Fig 4B, Data Supplement, Table S3).

## DISCUSSION

Our algorithmic approach integrates multiomics data, including genomic alterations, TMB, and immune gene expression profiles, to predict MSI status independent of direct assessment of microsatellite sites, particularly in cases where microsatellite sequencing is indeterminate. This approach leverages molecular data generated through routine NGS testing, enabling MSI inference without additional assays or tissue consumption. In our cohort of cases indeterminate by NGS, all cases predicted as likely MSI-H by the algorithm showed either loss of MMR protein expression by IHC or evidence of instability in partial microsatellite sequencing. We propose that any algorithmically inferred MSI-H cases should undergo follow-up testing, with MMR IHC serving as a practical and validated confirmatory approach.

Feature selection via Boruta algorithm identified key genomic and transcriptomic features predictive of MSI-H status, with many relevant to known MSI pathways (Fig 1), including alterations in *RNF43*, *ARID1A*, and core MMR genes such as *MSH2*, *MSH6*, and *MLH1*. Notably, *PMS2* was absent from the top-ranked features, which may reflect challenges in accurately detecting *PMS2* mutations due to interference from the *PMS2CL* pseudogene. *PMS2CL* shares high sequence homology with *PMS2* exons 11-15, often leading to false-positive truncating calls in MSS tumors.^19,20^ The prominence of *RNF43*—a gene frequently mutated in MSI-H colorectal and endometrial cancers—supports the biological validity of the model's feature selection.^21,22^

In contrast to established computational MSI-detection tools such as MSIsensor2, MiMSI, MSINGB, and fraction-based approaches, which rely on read-level analysis of defined microsatellite loci, our model differs by inferring MSI status without direct microsatellite sequencing. Because these locus-based algorithmic methods remain dependent on microsatellite sequencing itself, such approaches may yield indeterminate or unreliable results if coverage is inadequate across these regions. Thus, our approach represents an orthogonal strategy for MSI detection.

Of note, our study includes the use of two different transcriptomic platforms: (1) an amplicon-based panel (OIRRA) for the internal cohorts and (2) a whole-transcriptomic RNA-seq approach for external TCGA samples. Importantly, the internal data were used exclusively during model training and primary validation, and external data were used to assess cross-platform generalizability. Although platform-specific differences may exist, our results suggest that gene expression values are aligned across both methods. Moreover, despite being trained on CRC cases, our algorithm successfully predicted MSI status for both uterine and gastric cancers as well, providing evidence for a generalizable MSI-associated tumor microenvironment independent of site of origin. Similar to colorectal and gastric cancers, MSI is a critical biomarker in uterine cancer, aiding in both therapeutic decision making, as patients with recurrent or metastatic disease may benefit from ICIs,^23,24^ and for hereditary cancer risk assessment, as universal tumor testing identifies women with Lynch syndrome.^25,26^ The cross-tumor predictive capacity of our algorithm indicates a large degree of molecular overlap in genomic and immune gene expression profiles, highlighting the potential for pan-cancer MSI prediction capabilities, with the potential for improved immunotherapy response selection.^27^ These findings support MSI as a tumor-agnostic biomarker,^28^ in addition to demonstrating the potential clinical utility of multiomics approaches in precision oncology testing.

We applied our algorithm to cases with indeterminate MSI status due to microsatellite sequencing that did not meet quality criteria, identifying likely MSI-H cases for potential confirmatory follow-up testing. Accurate MSI testing is clinically important, particularly in CRC, because MSI-H status directly informs diagnosis,^29,30^ prognosis,^31,32^ hereditary cancer risk assessment, and treatment decisions.^33,34^ Recent evidence demonstrating high rates of clinical complete response with neoadjuvant dostarlimab in dMMR/MSI-H locally advanced rectal cancer further highlights the importance of accurate MSI classification for therapeutic decision making.^35,36^ Failure to identify MSI-H cases may result in patients being inappropriately excluded from effective immunotherapy options.^37^

Despite substantial overall performance, our study has several limitations. First, although our algorithm does not require microsatellite sequencing results to flag cases as likely MSI-H, the models were trained using high-confidence NGS MSI calls as ground truth, and cross-assay benchmarking against PCR or IHC was not available at scale for the internal cohort. As a result, assay-specific bias cannot be fully excluded and warrants evaluation in future studies. Second, data imputation was necessary to enable model prediction. Although failure of microsatellite sequencing may reflect suboptimal sequencing globally, a quality control review showed that other assay components, particularly RNA-seq, often retained passing or partially passing average quality metrics (Data Supplement, Table S3), in keeping with the known relative challenges associated with microsatellite regions. Third, validation of the MSI-indeterminate cohort was limited to cases with MMR IHC results available from surgical pathology reports. Because IHC remains the preferred initial test for assessing MSI-H/dMMR status in routine clinical practice, such data were available for a substantial number of cases.^38-41^ Fourth, as a reference laboratory, we do not routinely receive information regarding prior therapies, precluding assessment of how such treatments may influence the molecular features used for model training and evaluation.

Overall, the ML algorithm described in this study provides a clinically relevant approach for identifying likely MSI-H tumors and facilitating confirmatory testing in cases where NGS-based microsatellite sequencing is indeterminate. By leveraging multiomic data generated through routine clinical workflows, this method has the potential to improve diagnostic completeness by reducing missed opportunities to identify patients with MSI-H tumors. Future studies incorporating larger, prospective cohorts and expanded orthogonal validation may help further define the performance of this and similar algorithms across tumor types and clinical contexts.

## Data Availability

A data sharing statement provided by the authors is available with this article at DOI https://doi.org/10.1200/CCI-25-00367.
